# The Vigor of Defense Against Non-Self: Potential Superiority of Allorestricted T Cells in Immunotherapy of Cancer?

**DOI:** 10.3389/fonc.2013.00100

**Published:** 2013-05-03

**Authors:** Stefan Burdach, Hans-Jochem Kolb

**Affiliations:** ^1^Laboratory of Transplantation Biology, Children’s Cancer Research Center and Department of Pediatrics, Kinderklinik München Schwabing, Technische Universität MünchenMünchen, Germany

**Keywords:** allorestricted T cells, immunotherapy of cancer, haploidentical transplantation, tumor therapy, cellular immunity, adaptive immunity, TCR/MHC evolution

## Abstract

Men and sharks are both jawed vertebrates at the top of the food chain. Sharks are the first extant to develop adaptive immunity preserved to man throughout jawed vertebrates. We hypothesize here, that T cell receptor/major histocompatibility complex (TCR/MHC) interactions developed as the defense mechanism of carnivors against takeover by their victims’ cells derived pathogens. Germline encoded TCR segments have been conserved in evolution, providing the MHC bias of TCR. Ancestor genes of MHC polymorphisms may have first developed as a mating preference system, that later in evolution provided host immune responses destroying infectious non-self, yet maintaining tolerance to self. Pathogens may thus have simultaneously selected for alloimmunity. Allorejection has been observed in sharks and men. Cannibalism is a common ecological interaction in the animal kingdom, especially prevalent in aquatic communities; it favors selection of intraspecies allo responses for defense of self integrity. Alloreactive T cells do not undergo negative selection of strong TCR/MHC interactions; thus, they react stronger than self-MHC recognizing T cells. High levels of genetic diversity at MHC genes play a critical role in protecting populations of vertebrate species from contagious cells displaying stemness and homing features, including cancer cells. Recognition of self-MHC fails especially in diseases, which predominantly arise with age and after the peak of reproduction, e.g., cancer. So far, the treatment of malignant disease with autologous T cells has widely failed. Allorecognition constitutes an extremely powerful mechanism in evolution, which may be employed in immunotherapy of cancer by MHC-disparate, e.g., haploidentical transplantation and consecutive treatment with T cells from the donor parents recognizing tumor selective peptides presented by the non-inherited haplotype on the tumor.

## What Do Men and Sharks have in Common?

They are both jawed vertebrates and at the top of the food chain. Sharks have achieved this by the most powerful jaws on the planet. They were the first big meat eaters (carnivores) in evolution. Because the sharks’ teeth are replaceable, they may grow. Sharks may use over 20,000 teeth in its lifetime (Rafferty, 2011). Thus, they represent the paradigm of gnathostomata (jawed vertebrates). It is obvious why these first big meat eaters in evolution were jawed, but why were the first meat eaters also the first to develop adaptive immunity in the animal kingdom? And what may men learn from sharks?

In essence, we hypothesize here, that T cell receptor/major histocompatibility complex (TCR/MHC) interactions developed as the defense mechanism of carnivors against takeover by their victims’ cells or merely the pathogens these cells may carry. Thus, TCR/MHC interaction represents an extremely powerful mechanism in evolution. Such successful mechanisms are to be employed in immunotherapy of cancer.

## Jawed Vertebrates were First Carnivors, Discriminating Self and Non-Self by Adaptive Cellular Immunity by Means of V(D)J Recombination

Herein, cognate recognition of auto- as well as allo-MHC is critical. Germline encoded TCR segments have been selected by evolution to promote recognition of MHC, i.e., the MHC bias of TCR (Scott-Browne et al., 2011). This may be the reason why, T cell receptor V(D)J recombination-based auto and allo-MHC recognition first evolved in the earliest gnathostomata rays and shark 450 million years ago (Litman et al., 2010) in an evolutionary time span estimated to be less than 20 million years (Marchalonis and Schluter, 1998). These huge carnivors were exposed to a tremendous increase in pathogen exposure necessitating the development of versatile recognition systems discriminating between pathogen containing and non-containing cells. Allo and auto-MHC recognition are both based on the inherent MHC bias of the TCR.

V(D)J recombination-based MHC recognition occurred by a horizontal transposon insertion of bacterial genes into the vertebrate genome (Agrawal et al., 1998). This insertion placed the recombinase activating genes RAG1 and RAG2 into an already existing non-rearranging V-like exon of an Ig-domain-containing gene that was regulating cell mediated cytotoxicity or phagocytosis (Kaufman, 2002; Van Den Berg et al., 2004). The result was a method of rearranging genes to create molecules that had a greater structural diversity than those supplied by the genome for the rest of the vertebrate body. This event for these reasons is so radical and unprecedented that it has been described as a biological “Big Bang” (Schluter et al., 1999). While there has been evolutionary modifications in mammals, even the most basal group of living gnathostomes, the elasmobranchii or cartilaginous fish, contain all its basic elements: MHC class I and class II, Ig, TCR chains α, β, γ, and δ (Rast et al., 1997), and RAG1 and RAG2 (Agrawal et al., 1998; Laird et al., 2000). In spite of extensive search, none of these elements have been found in the phylogenically earlier agnathia (such as lampreys and hagfish) (Roitt et al., 1998; Mayer et al., 2002; Flajnik and Du Pasquier, 2008).

However, there is no evidence that the MHC is derived directly from allorecognition systems occurring earlier in evolution in plants and invertebrates. Such previous allorecognition systems, such as FuHC (fusion/histocompatibility) in the urochordate *Botryllus* function *to defend the genetic integrity of the individual* by preventing intraspecific stem cell parasitism as well as self-fertilization and inbreeding (Laird et al., 2005). There is no evidence that these allorecognition systems are ancestors of MHC.

Rather, based on the synteny to olfactorial polymorphisms, it has been assumed, that *MHC polymorphisms* may have developed as *a mating preference system* (Potts et al., 1991). In addition, infectious agents selected for host immune responses that destroy infectious non-self, yet maintain tolerance to self. Retroviruses and other pathogens may thus have simultaneously selected for alloimmunity (Gould et al., 2004), because polymorphism was primarily *advantageous to the population and* maybe secondary advantageous to *the genetic integrity of the individual* by providing allorecognition and allorejection.

## What Came First: Alloreactivity or the “Conventional Immune Response” Directed Against Peptides Presented by Self-MHC?

To answer this question, it may be helpful to compare MHC and TCR variability: Precedence of MHC variability before TCR variability, would argue in favor of peptide recognition and the conventional immune response. In contrast, precedence of TCR variability before MHC variability would argue in favor of allo-MHC reactivity. In that case, invariant MHC would have been too primitive for versatile peptide binding. Kurosawa and Hashimoto (1997) argues that MHC variability came in before TCR variability, i.e., first, there was only one TCR. These assumptions are based on the postulate, that allorecognition is MHC dominant. However there is also evidence that allorecognition may be peptide dominant (Felix and Allen, 2007). Moreover, the recent discovery, that a single amino acid in the TCR CDR2 is conserved from shark to man and is critical for MHC recognition by TCR (Scott-Browne et al., 2011) would argue in favor of MHC recognition before peptide recognition. This finding underlines *Nils Jerne’s postulate of a germ line encoded, innate MHC bias of the random TCR repertoire*, located in MHC binding CDR1 and CDR2. Nevertheless, sharks do have a thymus and are capable of peptide selection (Roitt et al., 1998). However, γ/δ TCR is capable of recognizing antigen independent of MHC and is likely to have preceded α/β TCR. We conclude, that based on the inherent TCR/MHC affinity, these two systems have developed simultaneously (Table 1).

**Table 1:** **The Schwabing Carnivor Hypothesis of Superiority of MHC allorestricted T cells in Immunotherapy of Cancer**.

MHC polymorphisms may have first developed as a mating preference system, that later in evolution provided host immune responses destroying infectious non-self, yet maintaining tolerance to self
Pathogens may thus have simultaneously selected for alloimmunity
Alloreactive T cells do not undergo negative selection of strong TCR/MHC interactions; thus, they react stronger than self-MHC recognizing T cells
So far, the treatment of malignant disease with autologous T cells has widely failed
Allorecognition constitutes an extremely powerful mechanism in evolution, which may be employed in immunotherapy of cancer
Haploidentical transplantation and consecutive treatment with T cells from the donor parents recognize tumor selective peptides presented by the non-donor parent haplotype on the tumor

**Figure 1:**
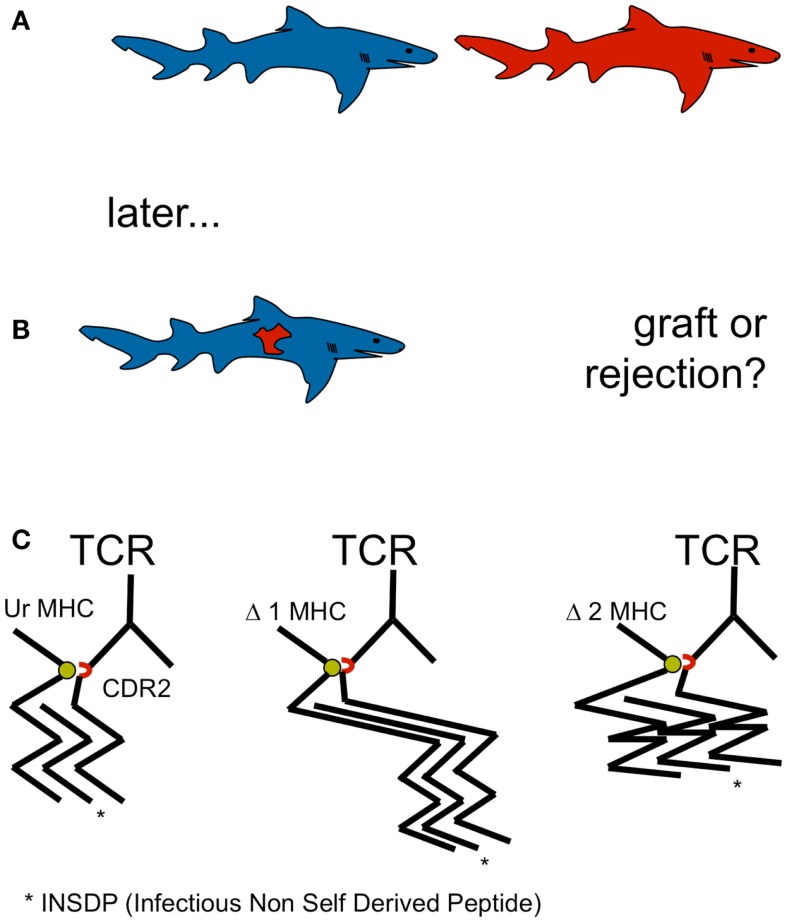
**(A,B)** Allorecognition by adaptive cellular immunity by means of V(D)J recombination is restricted to gnathostomata. Allo-MHC recognition may be essential in carnivorous vertebrates for self defense. Devouring of raw meat of the same species containing viruses and other parasites represents a selective pressure for MHC recognition and the development of allorejection. Since agnathan shark prey such as lamprey and hagfish did not display MHC, selective pressure for allorecognition to defend individual genetic integrity occurred first, when sharks started eating sharks: cannibalism became a constitutive feature of this extant. **(C)** A single amino acid in the TCR CDR2 (red), conserved from shark to man and critical for MHC recognition (green) by TCR argues in favor of MHC recognition before peptide recognition and underlines the postulate of a germ line encoded, innate MHC bias of the random TCR repertoire, located in MHC binding CDR1 and CDR2.

*Allorejection* has been *observed in* the rudimentary from of chronic graft rejection in elamosbranchs, including *sharks* (Kurosawa and Hashimoto, 1997). Allorejection has been observed in sharks and men, although it is not restricted to gnathostomata. However, allorecognition by adaptive cellular immunity by means of V(D)J recombination is restricted to gnathostomata. Thus, allo-MHC recognition may be essential in carnivorous vertebrates for self defense (Figure 1).

Of interest, while mechanisms of adaptive immunity are conserved in evolution from sharks to men, innate immunity displays great interspecies variation of mechanisms, although T and NK cells have both common ancestors and a common way to kill (granzyme/perforin). γ/δ T cells may bridge adaptive and innate immunity by combining adaptive features independent of MHC with rapid, innate-like responses (Vantourout and Hayday, 2013).

## Alloreactivity and the Schwabing Carnivor Hypothesis of Superiority of MHC Allorestricted T Cells in Immunotherapy of Cancer

Hitherto, the *selective pressure for allorecognition* has remained even more of an enigma for the scientific community than the mechanism allorecognition itself (Felix and Allen, 2007). Allorecognition has drawn the attention of immunologists in the context of transplantation, a relatively new phenomenon in evolution, not able to display selective pressure. However, *devouring of raw meat of the same species containing viruses and other parasites represents a selective pressure for MHC recognition and the development of allorejection*. Since agnathan shark prey such as lamprey and hagfish did not display MHC, selective pressure for allorecognition to defend individual genetic integrity occurred first, when sharks started eating sharks: cannibalism became a constitutive feature of this extant, but not restricted to it. In zoology, cannibalism is a common ecological interaction in the animal kingdom and has been recorded for more than 1500 species (Polis, 1981). It commonly occurs under natural conditions in a variety of species, including man (Elgar and Crespi, 1992) Cannibalism seems to be especially prevalent in aquatic communities, in which up to approximately 90% of the organisms engage in cannibalism at some point of the life cycle (Fox, 1975). Injuries violating epidermal and mucosal barriers during shark fights with ensuing cannibalism may have facilitated chimerism. Thus, it is not completely clear weather defense against prey derived pathogens alone or the prey itself as well was the driving force of development of adaptive immunity (Marchalonis and Schluter, 1998; Klimovich, 2002; Marchalonis et al., 2002). The consideration, that “there is a growing tendency to regard the evolutionary origin of adaptive immunity as being related to something other than defense against pathogenic microorganisms” has been attributed to Burnet himself (Rinkevich, 2004). Rationales of its development include the defense of host integrity against cells from the same or other species, and that it aided the management of symbiosis with commensals (Klimovich, 2002; McFall-Ngai, 2002).

Cellular adaptive immunity probably preceded humoral adaptive immunity in evolution (Parham, 1992). In jaw*less* vertebrates (agnathia), e.g., lamprey and hagfish, leucine-rich repeat variants (LRR) function as variable lymphoid receptors (VLR), the forerunners of T cell receptors and immunoglobulins (Rast et al., 1997). Adaptive cellular immunity based on V(D)J recombination and MHC recognition developed, according to our reasoning, first to protect the carnivor organism from foreign invasion by takeover by the cells of the victims of its cannibalism and/or from infection by the viruses, the devoured cells contained. Consecutively, the recognition of self-MHC by V(D)J recombination-based adaptive immunity may have developed simultaneously with allorecognition, both as critical mechanisms for defense of the biological ego against infection and non-self. In fact, *alloreactivity has much more in common with the conventional immune response* than previously thought.

Since *alloreactive T cells* did not undergo negative selection of strong TCR/MHC interactions, they *react stronger than self-MHC recognizing T cells*. Another hallmark of alloreactivity is the high precursor frequency of alloreactive T cells, which is 100-fold to 1,000-fold higher than the precursor frequency of T cells specific for any single foreign-peptide–self-MHC complex (Lindahl and Wilson, 1977). The high frequency of alloreactive T cells (1 in 10^3^–10^4^) enables their detection during a primary immune response, a defining feature of alloreactivity (Felix and Allen, 2007). Given the high frequency of alloreactive T cells may be of importance, as well, for immunotherapy with transgenic allorestricted T cells, since this population constitutes the target population of the transgene.

The *importance of alloreactivity for protection against non-self* is illustrated, e.g., by a transplantable tumor in the Tasmanian devil, a large carnivorous Australian marsupial. Transmission of this fatal clonal tumor by biting occurs due to depleted MHC diversity and threatens the devil with extinction (Siddle et al., 2007). High levels of genetic diversity at MHC genes play a critical role in protecting populations of vertebrate species from contagious cancer, of which two forms do exist, which are either transmitted by biting (as in the Devil Facial Tumor Disease, DFTD) or by sexual contact (as in case of the Canine Transmissible Venereal Tumor, CTVT, e.g., in Bernadine dogs) (Mello Martins et al., 2005). Species that have undergone genetic bottlenecks and have lost diversity at MHC genes are at risk of transmissible tumors. Moreover, evolution and selection for tumor variants capable of evading the immune response allow contagious cancers to cross MHC barriers (Belov, 2011). This could also apply to other cells displaying stemness and homing features after being devoured by carnivores (Table 1).

*In contrast to allorecognition, recognition of auto-MHC is not* triggering *effective* T cell responses *in all situations*, given the risks of autoimmunity. *Auto-MHC* responses fail especially in diseases, whose elimination is evolutionary not imperative, e.g., cancer. We perceive the co-evolution of tumor and host imprinted immuno-editing as the general cause of the failure of immune surveillance of cancer, yielding escape of the tumor from immune surveillance.

Thus far, the *treatment of malignant disease with autologous T cells has been effective only in part*; although a plethora of approaches has been developed including activation of the antitumor response through vaccination, promoting T cell function by leadfooting their accelerators and turning off their brakes, expanding existing tumor reactive T cells by adoptive T cell therapy, redirecting T cells utilizing TCR transduction, e.g., chimeric antigen receptor (CAR) engineering (Gao et al., 2013). Most advanced results are being seen in melanomas for the former (Restifo et al., 2012) and some in chronic lymphocytic leukemia and myeloma for the latter, i.e., the CAR approach (Maus and June, 2013). These advances may well be complemented and lead into a new dimension by employment of allorestricted T cells. The *success of allo-MHC recognition* by adaptive immunity both in terms of the evolutionary success of the defense of the biological ego in carnivorous vertebrates, as well as with respect to allorejection in MHC-disparate organ transplantation, *constitutes a rationale for employment of alloreactive T cells in antineoplastic immunotherapy* (Stauss, 1999) not only in hematopoietic malignancies (Kolb et al., 1990) but also in other mesenchymal neoplasias, such as sarcomas (Burdach et al., 2000) (Figure 1).

## Conclusion

In essence we postulate here, that TCR/MHC interactions developed as a defense mechanism of gnathostomata against takeover by their victims’ cells and/or their deleterious viral load. Allorecognition constitutes an extremely powerful mechanism in evolution, which may be employed in immunotherapy of cancer by e.g., haploidentical transplantation and consecutive treatment with T cells from the donor parents recognizing tumor selective peptides presented by the non-donor parent haplotype on the tumor. In addition, the application of alloreactivity, can be extended to mismatched unrelated transplants including umbilical cord blood transplants. Taken together, T cell reactivity toward allo-MHC, i.e., to inherited non-donor MHC alleles, can be advantageous in cancer therapy. This assumption may be dubbed the *Schwabing Carnivor Hypothesis of Superiority of MHC allorestricted T cells in Immunotherapy of Cancer (SCHySM)*.

## Conflict of Interest Statement

The authors declare that the research was conducted in the absence of any commercial or financial relationships that could be construed as a potential conflict of interest.
